# Implementing creative dance activities for primary school children to improve health and wellbeing: a qualitative study in the North East England

**DOI:** 10.1177/17579139241282549

**Published:** 2024-10-14

**Authors:** P van der Graaf, L Azevedo, C El Zerbi, PN Landindome, P Watson

**Affiliations:** Northumbria University, B117, Coach Lane Campus (West), Newcastle upon Tyne NE1 8ST, UK; Sheffield Hallam University, Sheffield, UK; Newcastle University, Newcastle upon Tyne, UK; Teesside University, Middlesbrough, UK; Teesside University, Middlesbrough, UK

**Keywords:** dance interventions, children, mental health and wellbeing, implementation, evaluation

## Abstract

**Aims:**

Evidence suggests that group arts activities with children build resilience and positive mental wellbeing. However, insufficient attention has been paid to how such activities can be implemented in practice across different contexts, particularly in socio-economically disadvantaged areas. Therefore, we explored the implementation of a dance-based intervention in two primary schools situated in an area of high economic deprivation in North East England.

**Methods:**

Our study explored Year 1 (age 5–6) and Year 5 (age 9–10) children, their parents, teachers and dance artists’ views of a creative dance intervention (South Tees Arts project; STAR) in two schools in North East England, using interviews and focus groups, combined with innovative data capture activities (i.e. movement activities, graffiti walls, songs and Vox Pops).

**Results:**

Children felt that STAR contributed positively to their emotional wellbeing and physical health. Teachers noticed improved confidence, engagement, literacy, and social and motor skills and less disruptive behaviour in class. Benefits continued beyond school, with children exercising at home to practice their dance moves. Several implementation barriers were identified ranging from limited time, large classes, dealing with challenging behaviours, the impact of COVID-19, stigma and anxiety. In response to these challenges, several solutions were developed during project delivery, such as artists and children working in pairs and role modelling by teachers and dance artists.

**Conclusions:**

We found three underlying mechanisms for successful implementation: (1) constant communication between teachers, dance artists and parents was essential to managing challenging behaviours, building personal relationships with children, and helping parents to get involved and support their children at home. (2) Linking dance activities to the school curriculum (using narratives from existing reading schemes) helped to support skill gaps. (3) A strong ethos of partnership between school, dance and arts providers and researchers ensured the adaptability and flexibility of projects.

## Introduction

Engaging children in creative activities such as dance has been recognised as an important health-promoting strategy that can improve their health and wellbeing, and promote social inclusion and positive environments.^1,2^ Over the last two decades, several creative public health interventions have been developed to support resilience and mental wellbeing in children and young people, such as dance mat exergaming.^3^ The latter is a combination of a computer game and physical activity in which dance steps are projected onto a wall or screen and players follow them on foot-activated floor pads. An umbrella review conducted by McCrary et al.^4^ highlighted that performing arts were the most popular form of arts participation with beneficial effects for children, adolescents, adults and older adults across 17 health domains (including body composition; cognitive; mental health; physical fitness; physical function; self-reported health/wellbeing; social functioning). These benefits occurred after as little as 30–60 min of dance per week through expressive (ballroom, social) and exercise-based (aerobic dance, Zumba) activities. However, only 8 out of 109 interventions listed in their review targeted children, and only one creative dance intervention (for older adults) was included. They also concluded that despite increasing evidence on the health benefits of arts engagement and participation, specific benefits associated with optimal modes and ‘doses’ of arts participation remain unclear, limiting evidence-based recommendations.

In one of the few studies that examined arts-based activities for children and young people,^5^ the authors concluded that structured group arts activities might help build resilience and contribute to positive mental wellbeing. However, they emphasised that the research evidence is limited and that insufficient attention has been paid to the context in which the activities took place and the mechanisms underpinning the use of the arts as an intervention.

Mansfield et al.^6^ confirmed the low quality of evidence on dance interventions that enhance subjective wellbeing for young people, with some evidence from grey literature identifying that it might promote negative feelings of incompetency and incapability.

Therefore, there is a need for more research that investigates the impact of dance activities on children’s mental health and wellbeing while taking into account the context in which these activities have been implemented to identify underpinning mechanisms that address potentially negative feelings. There is a particular paucity of evidence on the potential impact of school-based dance activities on young people living in areas of high economic deprivation, who are more likely to experience difficulties in emotional and social development.^7,8^ This can impact their academic achievement, and mental health and wellbeing in adult life,^9^ while having less access to mental health services and support than their peers living in more affluent areas.^10^ Studies conducted in the UK focused on dance interventions with secondary school girls and failed to increase physical activity levels, although the participants enjoyed the activities.^11^ Nevertheless, we could not find any published UK studies of dance activities in primary school children with mixed gender classes, which highlights a research gap that we are trying to address with our study.

Consequently, the aim of this study is to report on the implementation of a dance intervention (the South Tees Arts project; ‘STAR’) in two primary schools located in areas of high economic deprivation in North East England, and the perceptions of children, teachers, dance artists and project leads about the impact of the intervention on children’s health and wellbeing. Our study also considers the context in which the activities were implemented and identifies the underlying mechanisms of the intervention that supported children in overcoming challenges experienced in this context. Relevance of context and the need for mechanisms to address barriers experienced in this context emerged as key themes in our data analysis. We did not set out to conduct a realist study; however, the findings fitted well within this framework, and hence, we presented them in this configuration.

### Introducing the dance arts project

The project, named STAR, was a partnership between Northern Ballet, the North East and North Cumbria’s Child Health and Wellbeing Network, and community organisation TIN Arts, which aimed to find creative ways for these children to express themselves and to offer opportunities to access arts events that would benefit their health and wellbeing and reduce health inequalities.

Children in Year 1 (age 5–6) and Year 5 (age 9–10) from two primary schools located in Redcar and Middlesbrough took part in the study. The schools were located in the 20% of the most deprived districts/unitary authorities in England, as measured by the UK Index of Multiple Deprivation,^12^ with 25.2% of children living in low-income families and 22.1% of children classified as obese in Year 6.^13^

Children at these schools took part in weekly dance sessions during term time for the school year 2021–2022, with sessions lasting 30 min (with Year 1) to 45 min (with Year 5). The sessions explored engaging ways to help the children develop their social, emotional and physical skills by relating them to characters in a narrative ballet (Pinocchio). The dance artists encouraged creative expression and ownership of movements based on games or facilitated tasks. Content overlapped with the school curriculum, with dance activities drawing on the content of children’s lessons, for example, maths, cookery recipes, the solar system, and the numbering and naming of different body parts. The dance project also incorporated an arts award, for which the children built up a portfolio of evidence during their engagement with the project. Children also attended a performance of Pinocchio in a local theatre, and the dance project culminated with a celebratory performance of the pupils’ work at a local theatre venue, which brought all participating children and families together.

## Methods

We used a qualitative design, combining interviews and focus groups involving dance artists, teachers and project leads, with innovative data collection methods allowing children to explore their views about the development and implementation of the STAR project. In total, 37 participants were interviewed, individually or as part of focus groups, and creative engagement activities were run across the 2 primary schools, as summarised in Table 1.

**Table 1.** Sample of children, teachers, artists and project leads participating in focus groups, interviews and engagement activities (*n* = 37).

	Children year 1	Children year 5	Teachers year 1	Teachers year 5	Dance artists	Project leads
*School A*	6	3	3	2 + 1 follow-up	2 + 2 follow-up	3
*School B*	7	3	2	2 + 1 follow-up		
*Total*	13	6	5	4 (+ 2)	2 (+ 2)	3
*Timeline*	March and July 2022		December 2021–February 2022/July–October 2022		In November 2021; July 2022	August–September 2022
*Data*	Transcripts, photos, observation notes		Transcripts	Transcripts	Transcripts	Transcripts

### Data collection with children

Focus groups were conducted with 19 children from Years 1 (*n* = 13) and 5 (*n* = 6) between March and July 2022. Children were selected by propulsive sampling. Teachers from each Year group identified children with a range of individual characteristics (i.e. gender and ethnicity) to take part in the study. Two members of the research team (P.W. and P.N.L.) visited both schools to conduct the focus groups with children, incorporating, where possible, creative methods, such as movement activities, graffiti walls, songs and Vox Pops (further information about the activities are provided in Supplementary file 1). Focus groups explored pupils’ expectations, experience and access to arts, including their reflections on the implementation of the dance project at their school. In addition, the researchers observed two of the weekly dance sessions in each Year group for each school and took structured fieldnotes.

### Data collection with teachers, dance artists and project leads

Nine teachers from both schools participated in face-to-face focus group discussions before the intervention started (December 2021 and February 2022), facilitated by three members of the research team (P.W., P.N.L., L.A.). Focus groups with teachers investigated the acceptability, adoption and appropriateness of the dance project. Two follow-up interviews were conducted by L.A. with Year 5 teachers, one at each school, after completion of the STAR project (July and October 2022) to reflect on observed outcomes for children. These follow-up interviews were conducted online using MS Teams.

Two dance artists from TIN Arts were interviewed twice by C.E. at the start and end of the STAR project (in November 2021 and July 2022, respectively). These interviews focused on the artists’ views and experiences of the delivery, engagement, uptake, challenges and sustainability of the dance project. Follow-up interviews focused on outcomes and future development, and were conducted using MS Teams.

Finally, the three project leads (Executive Director of TIN Arts, Programme Lead for North East and North Cumbria Child Health and Wellbeing Network (NENC CHWN), and Director of Learning at Northern Ballet) were interviewed by P.N.L. between August and September 2022. The project leads represented the three partner organisations that developed and delivered the project, with TIN Arts providing overall project management and North Ballet providing the dance artists to the schools, while NENC CHWN resourced the evaluation. The interviews reflected on the planning and delivery stages of STAR and the impact of the project on the targeted audience. Interviews lasted between 25 min and 1.5 h (average of 45 min).

All focus groups, interviews and engagement activities were audio-recorded, and photos were taken of the graffiti walls and movement activities for data analysis.

Ethical approval for the study was granted by the Teesside University Research Ethics and Governance Committee (No 290/18).

Parental consent for children’s participation in the focus groups and engagement activities was obtained via letters to the parents sent home by both schools and through digital requests using the schools’ online Parent Mail system. Not providing consent did not preclude children from taking part in the dance activities. In addition, researchers attended two parent meetings, one at each school. Written consent for the interviews was also obtained from participating teachers, artists and project leads.

### Data analysis

Focus groups and individual interviews were transcribed verbatim. Qualitative data from focus groups and the individual interviews were analysed in NVivo 12 using an inductive approach to thematic data analysis described by Braun and Clarke.^14^ Following familiarisation with the data, line-by-line codes for each transcript were created along with extensive analytical memos. Coding continued until a sense of no new codes could be identified from the transcripts. Codes were then mapped and developed into main themes, which were named and defined.

Initially, research team members developed and drafted themes for each participant group separately. Final themes were discussed and interpreted by the research team in conjunction with their own fieldnotes, and illustrative extracts were discussed and selected for each theme. Themes are described narratively alongside the quotes which illustrate each theme. Quotes are referenced by the number assigned to each participant in the dataset to preserve their anonymity, and we have added a qualifier to each quote to identify participant group (e.g. child, teacher or artist). In addition, detailed fieldnotes were analysed from both the observations (i.e. dance sessions) and focus groups to add to the description and interpretation of identified themes.

## Results

We first describe the outcomes identified by children, artists, teachers and project leads, followed by reflections on four barriers in the implementation of dance activities in areas affected by high levels of economic deprivation. Finally, we outline the practical solutions developed by participants in response to these barriers, which are summarised in a model.

### Positive engagement skills development and mental health

Findings from the focus groups, interviews and engagement activities demonstrated positive engagement of children in both schools. Teachers reported that children were enthusiastic about participating in the project.

Artists felt that the dance activities and performances contributed to children’s creative, social, cognitive and physical skills, and increased their confidence. The focus group and engagement activities with children confirmed that STAR contributed to their emotional wellbeing as well as their physical health, with outcomes such as ‘Feeling more confident’, ‘More fit and well’ and ‘Full of energy’, most often mentioned by children in both schools across Years 1 and 5 These answers were recorded from the engagement activities with children, such as graffiti walls, songs and Vox Pops, described in the Methods section and Supplementary File 1:

*Yes, they love it. They’re devastated if it’s not on. They’re like, every Monday they come in, ‘Are we dancing today, are we dancing today, what time are we going to dance?’. So, they love it and it is really good for them, it is.* (Teacher, ID02)

Expressing themselves through dance appeared to help children feel more confident and happier to engage in schoolwork and other daily activities. Teachers noticed that children were more able to listen and engage in classwork, showing less disruptive behaviour. In addition, teachers reported improvements in children’s literacy skills:

*We can say that they’re more engaged and we can say that they seem to be more expressive in classroom when they’re doing role playing activities. They’re able to describe like the way they move, people move. They’re able to describe expressions better.* (Teacher, ID02)

Children mentioned feeling fitter and healthier, which continued beyond school. They reported doing more exercises at home and were keen to continue practising the dance moves learnt at school. Finally, project partners worked well together to deliver the STAR project within time and budget.

**Figure 1:**
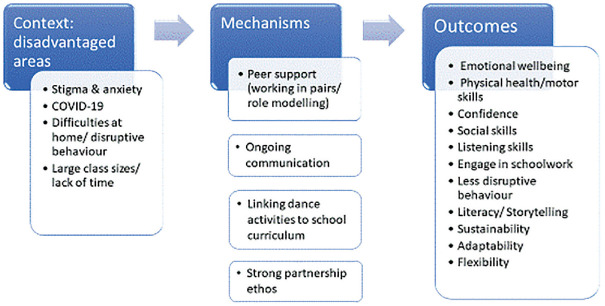
Model of context challenges, addressed by underlying project mechanisms, resulting in outcomes

In summary, STAR supported children in getting out of the classroom to experience a different learning style that helped with their mental health and emotional wellbeing. Teachers often do not have time or the tools to deal with mental health issues, particularly after COVID-19; the STAR project provided an outlet for children and an opportunity for teachers to address mental health issues by tapping into their creativity. Overall, children, teachers and dance artists were optimistic about the success of the project and suggested that the project should be delivered at a scale. The range of outcomes noted by the children, teachers and dance artists is summarised in the right-hand column of Figure 1.

### Barriers and solutions

Children, teachers, dance artists and project leads identified five barriers to engaging children in the dance activities and performances but also developed solutions in response to implementation challenges. We discuss each barrier, and the solution participants developed in response, separately below. We situated these barriers within the context of the schools being located within under-resourced communities, as these barriers related to entrenched problems experienced in these communities (see Figure 1).

#### Limited time and large classes

Both artists and teachers reported that two-weekly, 1-h sessions were deemed insufficient to engage with the children on a personal level, to get to know them and tailor the project to their needs, despite artists’ (and teachers’) best efforts to build relationships with the children. The ability to engage children in the dance activities was further hampered by the number of children that artists had to manage in each session, approximately 30 children at a time, reducing opportunities for personal contact with each child to build report and tailor the activities to their needs and creativity:

*It was about kind of trying to connect to the children and to see them, to be able to see them individually a bit and that’s hard when you’ve got, you know, twenty children in a space and you’re moving. So you’re trying to kind of manage the space and managing level noise levels and all the needs.* (Artist, follow-up interview, ID03)

The two dance artists worked together to deliver the dance sessions and, in doing so, were able to support each other with the limited session duration and large class sizes. In addition, observations of dance sessions and interviews with teachers highlighted that teachers in Year 1 often took part in the dance activities themselves to model the activities for their children.

#### Dealing with disruption

Teachers and staff had to spend considerable time dealing with disruptive behaviour from some children who, for various reasons, were not engaging in the dance activities. Children noted how disruptive behaviour from some of their classmates negatively impacted their own engagement, ‘spoiling the sessions’ for them. Some children also struggled to make the transition back to the classroom after the dance activities, finding it a challenge to cool and calm down and settle into the class.

Teachers helped dance artists to tailor sessions to personal needs and interests of children by sharing their knowledge about children with the artists, and by providing additional support and reassurance to children who felt less confident or anxious. In return, the successful engagement from children in the dance sessions reduced disruptive behaviour from children in the classroom:

*We’re starting to work with the teachers as well to try and understand some of the children. Whether it’s, you know, they have some diagnosis, behavioural or home life, so we’re starting to work with the teachers to understand that. And if they’ve got methods that they put in place with that child, whether there’s any trigger points.* (Artist, ID03)

#### Impact of COVID-19

The pandemic had a major impact on the ability of artists to deliver the dance activities in the schools, causing delays in project delivery. Teachers also commented on the impact of COVID-19, particularly the lockdowns, on children’s confidence and social skills, with children having missed 2 years of socialising and interacting with other children and adults. This meant that the children started from an even more disadvantaged position than they were already in when the STAR project was delivered in schools after the lockdown:

*I’m expecting these children to be year fives, I’m expecting them to be like ten year olds but really their last full year in school was year two. So they were, you know, seven year olds and then they’d missed these two years of socialising.* (Teacher, follow-up interview, ID10)

Children working in pairs were an important enabler, as more confident children supported the less confident, and motivated them to engage in the dance activities:

*So there were some who really threw themselves into it but some that surprised me. I have a boy in my class, he’s got a quirky personality, he’s, you know, very unique, but he’s sort of reserved and keeps to himself. He has to be quite confident to let his personality shine. But he threw everything into the dance, he was the one that came out with the energy, really challenged himself with the balances, and that was great.* (Teacher, follow-up ID10)

#### Stigma and anxiety

Artists and teachers commented on having to overcome gender stigma around dance activities. Boys in Year 5 appeared more self-conscious about engaging in dance activities, with some experiencing stigma (exhibiting embarrassment) and feeling nervous about taking part. In addition, teachers reported that some children experienced anxiety about going on stage in front of their peers and families during their theatre performance:

*Initially, when we introduced that we were going to be working with dance, the boys do, do the, argh, kind of we don’t want to do ballet, we don’t want to do dance. And they don’t automatically see it as a way of getting your feelings and your emotions out and a way of communicating. They don’t see it like that. They see it as someone who wears a dress.* (Teacher, ID03)

The stigma associated with dance experienced by some boys in Year 5 was overcome by inviting a professional male dancer into the schools to speak with the children about the important role of dance in his life, which helped to normalise the dance activities for boys.

Parents also commented to teachers on how they appreciated their children being publicly acknowledged and celebrated for their achievements. This does not happen very often for children who struggle with a formal curriculum which focuses on the assessment of highly structured subject knowledge and skills. Instead, the STAR project celebrated giving it a go and having fun, offering an alternative format and platform for learning:

*. . . if their kids aren’t great at Maths and English, again they’re not getting that celebration from their parents, that moment of being, you know, you were amazing and we’re just really proud of you. [. . .] Our big moments aren’t always fun ones, it’s a test at the end of half term or it’s, you know, like something negative is a big judgement moment. But that was like a real fun celebration sort of and to have that with the parents was really great. You could see how proud they were and how excited they were to go into an environment that they haven’t been in and watch their children do something really different, that was lovely.* (Teacher, follow-up interview, ID10)

## Discussion

### Key findings and relation to current literature

Our study contributes to the emerging evidence base about the impact of dance activities on the mental health and wellbeing of children. In line with other studies, our results showed that creative dance activities can improve children’s cognitive skills, mental health, and physical and social functioning.^4^ Children felt that STAR contributed to their physical and emotional wellbeing and that benefits continued beyond school, with children practicing at home. Expressing themselves through dance has the potential to help children feel more confident and happier to engage in schoolwork and other daily activities, though precisely how, and for whom, warrants further investigation. Teachers also noticed that children were more able to listen and engage in classwork, showing less disruptive behaviour and noted improvements in children’s literacy, social and motor skills.

Moreover, our study demonstrated that an understanding of context in which these activities have been implemented, such as areas of high economic deprivation, is crucial for addressing particular challenges faced by dance artists and teachers. This includes a lack of familiarity with the arts and stigma and anxiety about the arts and performance as experienced by some of the children in this study. The difficulties they face in their home situation and the disruptive behaviour this causes in classes, according to the teachers, were exacerbated by COVID-19. Further challenges included large class sizes and lack of time available to staff to build up trusting relationships with children to personalise their engagement. Similar issues were highlighted in the review of Zarobe and Bungay,^5^ who concluded that insufficient attention had been paid to the context in which art and dance activities took place and the mechanisms underpinning the use of the arts as interventions. Addressing the negative feelings towards dance experienced by some children living in disadvantaged areas is essential to produce positive health outcomes. As highlighted by Mansfield et al.,^6^ young people can experience negative feelings of incompetency and incapability towards the arts, and dance activities and group-based or peer-supported activities have the potential to improve subjective wellbeing of young people. Our findings demonstrate that working in pairs can help children to overcome negative feelings and resolve their differences to make up new dance moves. Similarly, working in pairs between teachers and artists can support personal engagement, with teachers and artists acting as role models and tailoring activities.

### Underlying mechanisms

From the barriers and facilitators, three mechanisms were identified as crucial for the development and implementation of dance and art interventions in primary schools in economically disadvantaged areas.

#### Communication

Artists relied on information from teachers about children’s individual needs. This exchange of information was often crucial for managing disruptive behaviour and tailoring activities. Artists would have preferred more time for reflection after sessions to document issues and learning with teachers.

Teachers did not feel they had enough information at the start of the project to understand what the children would be engaging in and therefore felt limited in their ability to support children, and by extension artists. A more detailed and bespoke introduction session to teachers with the artists at the start of the project would facilitate clear communication about expectations from both teachers and artists, and how they could support each other during the project:

*Ongoing communication between schools, dance and arts providers, and parents would help the latter to keep informed and understand how they can support their children at home and get involved themselves. Providing children with simple instructions to increase their engagement and more information about the performances, familiarity with the theatre and opportunities to rehearse on stage could also help to reduce their anxiety about performing on a stage. This need links to wider literature suggesting the importance of accountability at all levels for the implementation of public health intervention in school, requiring involvement and engagement for different professions and stakeholders based on clear communication.* ^15^

#### Linking dance and art activities to the school curriculum

Initial fears from teachers that STAR would take time away from delivering an already cramped and challenging curriculum were alleviated by their observations that the project actually supported the delivery of the curriculum. Teachers noticed an increase in children’s attention in class, combined with improved confidence to express themselves, enhancing engagement and learning in the classroom. According to the teachers, STAR taught children to articulate themselves differently by teaching them how to tell a story (through dance and music) and enabling them to listen to music and discover different storytelling elements, such as rhythm, pace, pitch and lyrics. These new skills directly supported the formal curriculum, such as English, where storytelling is a key ingredient. Therefore, linking narratives in dance sessions to existing reading schemes in schools could further support embedding dance and arts activities in the school curriculum. This mechanism is in line with findings from a recent literature review on the sustainability of school-based public health interventions.^16^ The review highlights staff observing a positive impact on students’ engagement and wellbeing as a key motivator for being involved in the delivery, in spite of time and resource constraints, which are listed as a key barrier in the review. The authors suggest that adaptation to existing routines and embedding into the curriculum is an essential part of the sustainability process, enabling schools to move beyond separate, isolated interventions.^15^

#### Partnership working

Collaboration between artists, teachers and project leads supported the successful delivery of the STAR project despite the COVID-19 pandemic and the various challenges this represented. A strong ethos of partnership ensured the adaptability and flexibility of the project, although some partners were less involved and struggled to make links with different teams.

Assuring the sustainability and future roll-out of dance interventions across other schools requires strong, collaborative and distributed leadership. The partnership ethos on which this project has been built demonstrates the value of different organisations coming together and contributing to a common goal, adding value from different perspectives. The importance of commitment and support from senior leaders has been highlighted in the literature,^16^ with external support from the wider system identified as a key sustainability factor.^17^ The three mechanisms, illustrated in Figure 1, underpin the use of dance and art activities by addressing barriers around stigma, lack of confidence and capability, and enabling positive health and wellbeing outcomes for children. These mechanisms are important to consider for implementation of creative public health interventions in primary schools located in disadvantaged areas and could be further explored and tested in future research.

### Strengths and limitations of study

The qualitative data collected in focus groups, interviews with dance artists, teachers and parents, and engagement activities with children yielded rich insights into their experiences of the project. While we were able to interview all dance artists and teachers involved in the delivery of STAR in both schools at the start, we only managed to interview two teachers, one from each school, in the follow-up interviews, limiting insights on the changes in teachers’ perceptions over the course of the project.

We deliberately limited the number of children in each activity to six to maximise their engagement and support their interactions in each activity, which was time-intensive. However, by only having a small proportion of the children across both schools, different opinions from other children might have been missed and limiting comparisons between schools, age groups and gender of children. Children were also sampled by the teachers from each Year group, based on their individual characteristics, to identify a range of experiences. This might have introduced an element of bias, but we felt in this small exploratory study that teachers were best placed to judge the different experiences of children given the limited sample size.

Finally, we struggled to recruit parents for the focus groups in both schools, considerably limiting their input in the project. Despite several recruitment efforts through the schools’ newsletters, attending parents’ mornings after the Pinocchio performance, and teachers’ best efforts to encourage parents to attend, we completed one focus group with parents in Year 5.

While explorative in nature, our study provided a practical model of mechanisms to consider when developing and implementing dance and art interventions in other primary schools. The collaborative design of the study with the active involvement of children, teachers, dance artists and project leads provided a degree of validity and credibility of the results and their usefulness for other contexts.

## Conclusion

Our study provided qualitative evidence of the potential positive impact of dance activities for children in primary schools based in areas of high economic deprivation, who are more likely to experience emotional and social development difficulties that can impact academic achievement.

Our findings highlighted the potential positive impact that these activities can have on children’s cognitive skills, mental and physical wellbeing, and social functioning. We also demonstrated the importance of understanding the context in which dance and art activities were implemented and the particular challenges this created for dance artists and teachers. We identified three underlying mechanisms that need to be addressed to encourage sustained, flexible and personalised engagement from children in the dance activities.

Future research could focus on the long-term benefits of piloting interventions across a range of school settings.

## Supplemental Material

sj-docx-1-rsh-10.1177_17579139241282549 – Supplemental material for Implementing creative dance activities for primary school children to improve health and wellbeing: a qualitative study in the North East England Supplemental material, sj-docx-1-rsh-10.1177_17579139241282549 for Implementing creative dance activities for primary school children to improve health and wellbeing: a qualitative study in the North East England by P van der Graaf, L Azevedo, C El Zerbi, PN Landindome and P Watson in Perspectives in Public Health
