# Supplementary material for: Implementing creative dance activities for primary school children to improve health and wellbeing: a qualitative study in the North East England
Source: Perspect Public Health. 2024 Oct 14;144(5):304–11. doi: 10.1177/17579139241282549 (PMC11542315; doi:10.1177/17579139241282549)
Supplement: sj-docx-1-rsh-10.1177_17579139241282549 – Supplemental material for Implementing creative dance activities for primary school children to improve health and wellbeing: a qualitative study in the North East England [file sj-docx-1-rsh-10.1177_17579139241282549.docx]

# Supplementary file 1

##

## Topic guide for interviews with dance artists

| 1. **Background** | |
| --- | --- |
| Could you describe what happens in a dance session? | Probes:   1. Could you describe what you aim to do in a dance session? 2. How were these aims developed/derived? 3. Could you describe the activities involved in a dance session? 4. Is there a rationale or logic for selecting these activities in particular? |
| 1. **Process** | |
| What do you think works well in the dance sessions? | Probes:   1. What do you think doesn’t work so well? 2. What changes would you make to improve the dance sessions, and why? 3. What are the challenges you have encountered to delivering the dance sessions? |
| 1. **Evaluation** | |
| Can you describe the ways in which you can tell that the children have benefited from the dance sessions? | Probes:   1. How do you define a successful dance session? 2. How is success indicated to you? 3. How would you measure a successful session? 4. How can you tell if a dance session did not go as well as you had hoped? How is this indicated to you? 5. Do you make use of any particular methods to do this? 6. If yes, what are these methods and how are the children involved? 7. Do you think the methods you use to evaluate sessions are acceptable to the children? 8. What would you do to improve existing methods of evaluation be improved? |
| 1. **General** | |
| How do you think dance generally helps the children? | Probes:   1. What do you think the children learn from the sessions? 2. Can you talk about the strategies you use to engage different children to join in the sessions? 3. Could you describe instances in which you have found it difficult to engage the children in the sessions? 4. Could you talk about the skills you think the children gain or develop from participating in dance? 5. Have you noticed any changes in the children (e.g. in confidence, self-efficacy) from the start to the end of sessions? And over the course of the programme? |
| 1. **Health & Wellbeing** | |
| What do you think the health and well-being benefits are for children who participate in your dance sessions, if any? | Probes:   1. Could you describe the physical benefits? 2. Could you describe the mental health benefits? 3. Could you describe the social benefits? 4. Are there any other unexpected benefits you have noticed the children gain from taking part? 5. How do you recognise/measure these benefits? |

## Topic guide for interviews with teachers

1. How do you see the use of extra-curricular activities for promoting children’s wellbeing?

a. Could you describe some of the barriers?

b. Could you describe some of the key elements of the wellbeing activities which appears to work?

2. How do you see the use of dance and arts for promoting wellbeing in children?

a. What do you think are the mechanisms on how this would work?

b. Do you see any other benefits beyond wellbeing?

3. How do children engage with dance and arts in general?

a. What do you believe are children’s perceptions of dance and arts?

b. Can you identify any barriers or facilitators for engagement?

c. Which factors would affect engagement (e.g. gender, skills,…)?

4. How important is the engagement of families in the activities?

a. What would be the benefits for families?

b. What would be the barriers to family’s engagement?

c. How can we overcome these barriers?

5. How do you see this programme working in the longer term?

a. Could you see this being embedded in the curriculum?

b. How willing are you to receive training to deliver this programme?

c. How do you want the training to be delivered?

6. How would one be able to tell if a programme had a positive effect on children’s wellbeing?

a. Have you noticed any changes in behaviour and attitudes?

b. What would be positive changes you would like to see in the short and longer-term?

c. How do you see the use of the academic evaluation to explore children’s views on the programme and to examine the effect on children’s health and wellbeing?

d. Are there any challenges? How can we overcome those?

e. What are some of the selling points of an academic evaluation?

## Topic guide for focus groups and engagement activities with children

**Examples of engagement activities:**

- Children were asked to run to a corner in the class room with a photo that best represented their answer to the question asked by the researchers.
- Graffiti walls consisted of flipcharts on a table that children could stick post-it notes on with their answers to questions highlighted on the flipchart.
- VoxPops (from Vox populi, a Latin phrase that literally means "voice of the people") applied a journalism technique by giving children a microphone to do mini-interviews, with children passing the microphone to each other to record their responses to questions about the STAR project asked by the researchers.
- One of the researchers (PNL) also wrote a song, asking children to join in with hand and body signals to respond to questions about the project.

We developed and piloted these engagements methods to engage the children in the research in a fun and interactive way to elicit multiple responses to our focus group question. All piloted methods were well received by the children; the VoxPops in particular proved popular with children in both Year groups being excited to hold the microphone and share their experiences.

**Warm up**

A bit about what we are going to be doing and why. Any questions?

**A bit about you – EXERCISE – get them moving**

| Questions | Methods |
| --- | --- |
| **About you**   - Do you live in Grangetown/Thorntree? - Do you like where you live? If yes why and if no, why not? - Do you walk to school or come by car? - How long does it take you to get to school? - Do you have brothers and/or sisters? | **Yes/No Corners**  **General discussion** |
| **General health and wellbeing**   - Do you think you are fit and healthy? - What do you do that you think keeps you fit? - How many times do you exercise? - What type of exercises do you do? - General thoughts on how they feel etc | **Yes & No flipchart sheets on the table – stick post-its on relevant sheets** |
| **The Dance Project** |  |
| - Who likes to dance? How/why – does it make you feel happy, healthy, sad, tired? - Are you enjoying doing the dancing in school? - What do you like best about the dance project? - Is there anything you don’t like about the dance project? Why? | **Song** |
| **Physical/Emotional Wellbeing**   - Do you think the dance project helps you feel better about yourself? –prompts: more confident, wanting to exercise/dancing more, dancing with your friends - Have you started to do more exercise outside of school? - Do you think you can sleep better after any activity or exercise? - Have you made any changes in what you eat recently? Favourite meal? | **VoxPops**  **General discussion** |
| **Finishing off**   - One word to describe what you think about the dance project | **Post-its** |
